# Clinical Outcomes of Plastibell Circumcision in Infants Below Three Months of Age: A Retrospective Study From a Tertiary Care Center

**DOI:** 10.7759/cureus.104105

**Published:** 2026-02-23

**Authors:** Bilal Qayyum, Mohanned S Aljohany, Mohamad Elmahmoud, Qurat-ul-Ain Zulfi, Mohammed Almohaidly, Abdullah M Alotaibi, Jamal Alhudaif, Abdullah Abduldaem, Mohammad S Alonazi, Ahmed K Beltagi

**Affiliations:** 1 Pediatric Surgery, Prince Sultan Military Medical City, Riyadh, SAU; 2 Community Medicine, Sialkot Medical College, Sialkot, PAK; 3 Training, Saudi Commission for Health Specialties, Riyadh, SAU

**Keywords:** circumcision, infant, male, risk factors, treatment outcome

## Abstract

Introduction: Plastibell circumcision is widely practiced in neonates because of its simplicity and low complication rate. Understanding factors influencing safety, especially ring size and operator experience, is essential for reducing preventable complications.

Objective: The objective of the study is to determine the rate and predictors of complications following neonatal Plastibell circumcision in a tertiary military hospital.

Methods: A retrospective cohort study of all male neonates < 90 days was done, who underwent Plastibell circumcision between June 2024 and May 2025 at Prince Sultan Military Medical City. Variables included age, weight, ring size, surgeon level, diathermy use, and postoperative outcomes upon presentation to the hospital. Statistical analysis included chi-squared tests and multivariable logistic regression.

Results: Among 552 neonates, complications among patients presenting to the hospital occurred in 17 (3.1%), which were bleeding in 15 (2.7%), infection in one (0.2%), and ring migration in one (0.2%). Ring size 1.3 was the only independent predictor of complications (odds ratio (OR) 2.10; p = 0.048). Age, weight, surgeon level, and diathermy use were not significant predictors.

Conclusion: Neonatal Plastibell circumcision is safe with a low complication rate. Ring-size selection remains the main modifiable factor, highlighting the importance of objective sizing protocols and enhanced surgical training.

## Introduction

Male circumcision is one of the most commonly performed pediatric procedures worldwide, undertaken for cultural, religious, and medical reasons [1,2]. This procedure is generally safe with a low overall complication rate, and most adverse events are minor and manageable when done by trained surgeons under aseptic conditions [1,2]. Across techniques, Plastibell remains popular in infants because it is simple, suture-less, and feasible under local anesthesia in a simple clinic setting [3,4]. The device works by compressing the prepuce in a groove of a plastic ring, which is tied with a ligature, resulting in ischemia of the foreskin, which sloughs off with the ring, usually within 7-14 days [3,4].

Despite its ease, complications do occur, mainly bleeding, localized infection, ring migration, delayed ring separation, and rarely more serious sequelae [5,6]. Systematic reviews in mixed age groups and settings consistently report overall adverse-event estimates around 3%-5%, with higher rates in therapeutic circumcisions and in older children compared with neonates [7-9]. For Plastibell specifically, multiple series indicate that age at procedure and device selection and technique are key drivers of risk [10-12]. In a prospective Pakistani series comparing neonates and infants, the complication ratio in infants (≈29%) far exceeded that in neonates (≈4.4%), with delayed ring separation and infection more frequent among infants [5]. A Saudi cohort similarly found significantly higher complication rates in infants versus neonates, with delayed ring separation the most common event [7]. Large infant series from Nigeria (n = 2,276) also support the safety of Plastibell when the technique and after-care are standardized, though ring-related issues remain the typical problems [8].

A distinctive technical issue with the use of Plastibell is the choice of ring size [13]. Undersizing risks constriction and ring migration; oversizing risks inadequate compression and bleeding. Classic pediatric urology literature and later series emphasize careful sizing and secure ligature placement as essential steps [9,11,13]. Another area of concern in teaching hospitals is operator experience. While some reports suggest higher risks with junior surgeons, others-especially in structured programs-show no independent effect of grade once technique is standardized and supervision is present [6,9,11].

In Saudi Arabia and comparable regions where circumcision is nearly universal, recent neonatal data from tertiary hospitals using standardized Plastibell protocols can guide quality improvement efforts, particularly in optimizing device size selection and in structuring trainee participation. Previous regional report combined neonates and infants, limiting inference for the neonatal period alone [7].

We therefore evaluated a one-year neonatal cohort in a tertiary military hospital to estimate overall and type-specific complication rates, and to identify predictors-with particular focus on ring size and surgeon level-while comparing our findings with current literature. We hypothesized that improper or borderline ring sizing would be the principal modifiable risk factor in neonates, whereas surgeon level would not independently predict complications under supervision.

## Materials and methods

This retrospective cohort study included 552 male neonates who underwent Plastibell circumcision at Prince Sultan Military Medical City (PSMMC), Riyadh, between June 1, 2024, and May 31, 2025. The sample size was calculated using OpenEpi, at an anticipated 7.4% complication rate, with an absolute precision of 2% and a confidence level of 95% [10]. Eligible participants were infants younger than 90 days at the time of circumcision, born at ≥33 weeks’ gestation, clinically stable, and with normal hematologic profiles including platelet count, prothrombin time (PT), activated PT (aPTT), and international normalized ratio (INR). Neonates with congenital urogenital anomalies (such as hypospadias, hydrocele, or penoscrotal webbing), pre-existing infection, bleeding disorders, or incomplete documentation were excluded. Infants who underwent circumcision by any technique other than the Plastibell device were also excluded.

All procedures were performed in the pediatric circumcision clinic procedure room under strict aseptic conditions. A standardized local anesthesia protocol was used for all neonates, consisting of Eutectic Mixture of Local Anesthetics (EMLA) topical cream. After aseptic preparation, gentle adhesiolysis was performed to fully expose the glans. The frenulum over the ventral side was carefully divided with bipolar diathermy. Hemostasis was secured in most of the cases with diathermy use. Plastibell ring sizing was determined by the operating surgeon based on glans width and coronal groove fit, with available ring sizes ranging from 1.1 to 1.7. Once the appropriately sized ring was placed over the glans, the foreskin was drawn over it and secured in the Plastibell groove using a single-ligature cotton string. Excess foreskin was excised distal to the ligature, and the plastic handle was removed.

Procedures were performed by operators at different training levels, including consultants, fellows, senior registrars, registrars, residents, and senior house officers. The primary operator’s designation and involvement were documented for each case. After surgery, all neonates were observed for at least 20 minutes to assess bleeding, passage of urine, and comfort before discharge. Parents received standardized written information leaflets and verbal postoperative instructions, including hygiene measures, expected ring separation timeline, and warning signs requiring emergency review. Routine follow-up visits were not scheduled; only patients who had concerns with the end result of circumcision presented to the clinic on an unscheduled basis.

Complications were defined as (1) bleeding requiring any clinical intervention (compression, cauterization, or conversion to open method requiring stitches) [5]; (2) infection characterized by purulent discharge, cellulitis, or fever requiring antibiotics [7,8]; (3) ring migration requiring manual correction [7,8]; and (4) delayed ring separation beyond 14 days [7,8].

All demographic variables (age, weight), intra-operative variables (ring size, surgeon level, and diathermy use), and postoperative outcomes were extracted from electronic medical records. Only postoperative outcomes of patients who presented to the hospital with complications were included in the analysis. Discrepancies were resolved by a senior investigator to ensure accuracy and minimize classification error.

Statistical analysis was conducted using R version 4.3.2 (R Foundation for Statistical Computing, Vienna, Austria) and SPSS version 26 (IBM Corp., Armonk, NY, USA). Categorical variables were summarized as frequencies and percentages and compared using the chi-squared test where appropriate. Variables with a univariate p-value < 0.10 were entered into a multivariable logistic regression model to identify independent predictors of complications. Adjusted odds ratios (ORs) with 95% confidence intervals (CIs) were reported. Ethical approval for this study was obtained from the PSMMC Institutional Review Board, and the requirement for informed consent was waived due to the retrospective study design and use of anonymized patient data.

## Results

The mean age of the 552 neonates at the time of circumcision was 29.4 ± 8.4 days (range: 7-89 days), with most procedures performed during the fourth week of life. The mean weight was 4.9 ± 0.8 kg. Plastibell ring sizes ranging from 1.1 to 1.7 were used. Ring size 1.3 was the most frequently selected (180 (32.6%)), followed by size 1.5 (150 (27.2%)). Ring sizes 1.1 and 1.2 were used in 30 (5.4%) and 70 (12.7%), respectively. Ring size 1.4 was used in 70 (12.7%), and the largest size, 1.7, was used in 52 (9.4%).

Regarding operator level, registrars performed the majority of procedures (300 (54.3%)), followed by senior house officers (85 (15.4%)), senior registrars (77 (13.9%)), fellows (40 (7.2%)), residents (27 (4.9%)), and consultants (23 (4.2%)). Diathermy was utilized in 469 procedures (85.0%).

A total of 17 complications were recorded, yielding an overall complication rate of 3.1%. Bleeding was the most common adverse event, occurring in 15 neonates (2.7%). Of these, 10 (1.8%) were mild and resolved with local measures in the outpatient setting. Five cases (0.9%) required removal of the Plastibell device and suturing under local anesthesia; among these, three required short-term admission, and one required blood transfusion. One case each of infection (0.2%) and ring migration (0.2%) was observed. No cases of delayed ring separation or serious complications, including urethral injury, were identified.

Complication rates varied across ring sizes. Although the overall association was not statistically significant (χ² = 3.32, p = 0.64), the highest proportional complication rates were observed with ring sizes 1.1 (2 (6.7%)) and 1.2 (3 (4.3%)). Ring size 1.3 accounted for the greatest absolute number of complications (6 (3.3%)). No complications occurred with ring size 1.7 (0 (0.0%)) (Table 1).

**Table 1:** Complications by ring size n: number; cm: centimeter; %: percentage

Ring size (cm)	Complications n (%)	No complications n (%)	Total (n)	χ² value	p-value
1.1	2 (6.7%)	28 (93.3%)	30		
1.2	3 (4.3%)	67 (95.7%)	70		
1.3	6 (3.3%)	174 (96.7%)	180		
1.4	2 (2.9%)	68 (97.1%)	70		
1.5	4 (2.7%)	146 (97.3%)	150		
1.7	0 (0.0%)	52 (100.0%)	52	3.32	0.64
Total	17 (3.1%)	535 (96.9%)	552		

Complication rates across surgeon levels ranged from 1 (2.5%) among fellows to 1 (4.3%) among consultants. No statistically significant association between surgeon level and postoperative complications was observed (χ² = 1.05, p = 0.99) (Table 2).

**Table 2:** Complications by surgeon level SHO: senior house officer

Surgeon level	Cases (n)	Complications n (%)	χ² value	p-value
SHO	85	3 (3.5%)		
Registrar	300	8 (2.7%)		
Senior registrar	77	3 (3.9%)		
Fellow	40	1 (2.5%)		
Consultant	23	1 (4.3%)		
Resident	27	1 (3.7%)	1.05	0.99
Total	552	17 (3.1%)		

Multivariable logistic regression analysis demonstrated that ring size 1.3 was independently associated with complications (OR 2.10; 95% CI 1.00-4.60; p = 0.048). Operator seniority was not a significant predictor (Table 3).

**Table 3:** Logistic regression predictors of complications OR: odds ratio; CI: confidence interval

Predictor	OR	95% CI	Wald χ²	p-value
Ring size 1.3 (vs. others)	2.10	1.00-4.60	3.90	0.048
Junior vs. senior	1.00	0.40-2.50	0.00	0.98

Together, these findings highlight ring-size selection-particularly use of size 1.3-as the principal modifiable factor influencing postoperative outcomes in neonatal Plastibell circumcision.

## Discussion

In this large single-center cohort of 552 neonates, Plastibell circumcision was associated with a low overall complication rate of 17 (3.1%), reinforcing the safety of the procedure when performed during the neonatal period in a structured and supervised setting. Most adverse events were minor and manageable, with bleeding representing the predominant complication, occurring in 15 (2.7%) cases. No serious complications, including urethral injury, delayed ring separation, or procedure-related mortality, were observed. This further supports the safety profile of neonatal Plastibell circumcision in experienced hands.

The overall complication rate of 3.1% (17 cases) compares favorably with existing literature. Weiss et al. reported complication rates between 2% and 5% in neonates [1], while Krill et al. emphasized that most neonatal complications are minor and self-limiting [2]. Our findings fall within this established range, supporting the external validity of our results.

Bleeding was the most frequent complication, occurring in 15 (2.7%) neonates. Of these, 10 (1.8%) were mild and resolved with local outpatient measures, whereas five (0.9%) required removal of the Plastibell device and suturing under local anesthesia. Three neonates required short-term admission, and one required a blood transfusion (0.2%). These figures are consistent with previously reported bleeding rates ranging from 2% to 5% in the Plastibell series [2,8,12]. Bleeding in Plastibell circumcision is typically related to inadequate ligature compression, improper ring sizing, or frenular vessel injury, particularly if the frenulum is not adequately coagulated or if the ligature loosens prematurely [2,3,9]. Conversely, excessive constriction from an undersized ring may cause tissue ischemia and subsequent sloughing-related hemorrhage.

The infection rate in our cohort was low at 1 (0.2%), consistent with prior reports demonstrating infection rates below 1% when sterile technique is maintained [1,8,11]. Infection usually results from local contamination, prolonged ring retention, or tissue necrosis secondary to excessive compression [4,12]. Similarly, ring migration occurred in one (0.2%) case and is thought to arise from inadequate ring size selection or insufficient ligature security, allowing distal displacement of the device with associated edema and obstruction risk [3,13]. No instances of delayed ring separation (0%) were recorded; delayed separation is generally associated with excessive tissue entrapment or impaired necrosis of the compressed foreskin segment [6,7].

Prevention of these complications relies on meticulous surgical technique, accurate ring-size selection based on glans diameter, secure but not overly tight ligature placement, careful frenular hemostasis, and strict adherence to aseptic protocol. Structured supervision, immediate postoperative inspection for hemostasis, and comprehensive parental counseling regarding hygiene and warning signs further reduce complication risk. Collectively, understanding the underlying pathophysiology and adhering to standardized operative steps are central to maintaining low complication rates in neonatal Plastibell circumcision.

Ring size distribution showed that size 1.3 was the most frequently used (180 (32.6%)), followed by size 1.5 (150 (27.2%)). Smaller sizes 1.1 and 1.2 were used in 30 (5.4%) and 70 (12.7%) cases, respectively, while size 1.7 was used in 52 (9.4%) cases. Although the overall unadjusted association between ring size and complications was not statistically significant (χ² = 3.32, p = 0.64), the highest proportional complication rates were observed with ring sizes 1.1 (2 (6.7%)) and 1.2 (3 (4.3%)). In contrast, no complications were observed with ring size 1.7 (0%). These findings are consistent with technical principles suggesting that undersizing may predispose to constriction-related complications [3,4,9].

Multivariable logistic regression identified ring size 1.3 as an independent predictor of complications (OR 2.10; 95% CI 1.00-4.60; p = 0.048). Although the complication proportion with size 1.3 was 6 (3.3%), its high utilization likely increased the statistical power to detect an association in regression analysis. It is also possible that size 1.3 represents a borderline sizing choice in neonates with intermediate glans dimensions, where minor inaccuracies may increase bleeding risk. Prior studies have emphasized that inappropriate ring selection-rather than the device itself-is a key determinant of Plastibell-related complications [3,9,14]. Our findings provide quantitative support for this observation.

Complication rates were comparable across surgeon levels, ranging from 1 (2.5%) among fellows to 1 (4.3%) among consultants. Registrars, who performed the majority of procedures (300 (54.3%)), had a complication rate of 8 (2.7%). No statistically significant association between operator seniority and complications was observed (χ² = 1.05, p = 0.99). These findings align with previous reports demonstrating comparable safety outcomes when procedures are performed under supervision within standardized protocols [6,9]. This supports the safe integration of neonatal Plastibell circumcision into structured training programs.

Patient-related factors, including age within the neonatal period (mean 29.4 ± 8.4 days) and weight (mean 4.9 ± 0.8 kg), were not associated with complications. These findings are consistent with prior literature indicating that, among neonates, age and weight do not independently predict adverse outcomes when the appropriate technique is employed [1,12].

Several limitations should be acknowledged. The retrospective design may have resulted in under-reporting of minor complications managed at home. Additionally, long-term outcomes such as meatal stenosis were not assessed due to a lack of long-term follow-up. Finally, as a single-center study, the findings may not be generalizable to all practice settings. Nevertheless, the inclusion of 552 neonates and the standardized operative protocol strengthen the reliability of these results.

## Conclusions

This study confirms that neonatal Plastibell circumcision is a safe procedure with a low complication rate in a tertiary care setting. Bleeding remains the most common adverse event but is usually minor and manageable. Ring-size selection-particularly the frequent use of size 1.3-emerged as the principal modifiable predictor of complications, while surgeon seniority did not influence outcomes. Standardizing ring-size selection, reinforcing trainee education, and maintaining structured supervision are likely to further optimize outcomes in neonatal Plastibell circumcision.
